# Unexpected Anisotropy of the Electron and Hole Landé g-Factors in Perovskite CH_3_NH_3_PbI_3_ Polycrystalline Films

**DOI:** 10.3390/nano12091399

**Published:** 2022-04-19

**Authors:** Guadalupe Garcia-Arellano, Gaëlle Trippé-Allard, Thomas Campos, Frédérick Bernardot, Laurent Legrand, Damien Garrot, Emmanuelle Deleporte, Christophe Testelin, Maria Chamarro

**Affiliations:** 1 Institut des NanoSciences de Paris, CNRS, Sorbonne Université, F-75005 Paris, France; guadalupe1314@gmail.com (G.G.-A.); frederic.bernardot@insp.jussieu.fr (F.B.); christophe.testelin@insp.jussieu.fr (C.T.); maria.chamarro@insp.jussieu.fr (M.C.); 2 LuMIn (Laboratoire Lumière, Matière et Interfaces), CentraleSupélec, CNRS, ENS Paris-Saclay, Université Paris-Saclay, F-91190 Gif-sur-Yvette, France; gaelle.allard@universite-paris-saclay.fr (G.T.-A.); thomas.campos@ens-paris-saclay.fr (T.C.); emmanuelle.deleporte@ens-paris-saclay.fr (E.D.); 3 Institut Photovoltaïque d’Île-de-France (IPVF), F-91120 Palaiseau, France; 4 GEMaC (Groupe d’Etude de la Matière Condensée), CNRS, UVSQ, Université Paris-Saclay, F-78000 Versailles, France; damien.garrot@uvsq.fr

**Keywords:** halide perovskite, CH_3_NH_3_PbI_3_, g-factor, polycrystalline film, photo-induced Faraday rotation

## Abstract

In this work, we studied, at low temperature, the coherent evolution of the localized electron and hole spins in a polycrystalline film of CH_3_NH_3_PbI_3_ (MAPI) by using a picosecond-photo-induced Faraday rotation technique in an oblique magnetic field. We observed an unexpected anisotropy for the electron and hole spin. We determined the electron and hole Landé factors when the magnetic field was applied in the plane of the film and perpendicular to the exciting light, denoted as transverse ⊥  factors, and when the magnetic field was applied perpendicular to the film and parallel to the exciting light, denoted as parallel  ∥  factors. We obtained $\left|g_{e,⊥}\right|=2.600\;\pm \;0.004$, $\left|g_{e,∥}\right|=1.604\;\pm \;0.033$ for the electron and $\left|g_{h,⊥}\right|=0.406\;\pm \;0.002$, $\left|g_{h,∥}\right|=0.299\;\pm \;0.007$ for the hole. Possible origins of this anisotropy are discussed herein.

## 1. Introduction

In the last few years, lead halide perovskites have attracted great interest in the scientific community. This class of materials exhibits many excellent photo-physical properties, such as broad absorption spectra, tunable band gap emission, narrow line-width, high luminescence yields, large charge carrier mobilities, and dielectric constants. They are very useful for their potential applications in photovoltaic, opto-electronic, and electronic devices, such as light-emitting diodes or lasers, photodetectors, photo-sensors, and transistors [1,2,3,4,5,6,7,8,9]. In addition, these materials are also promising for opto-spintronic devices [10,11] because they show long spin lifetimes [12,13,14,15], chiral properties [16,17,18], and large Rashba splitting [19]. The synthesis of engineered nanostructures [20,21,22] extends the future applications of these materials to the domain of quantum optics and quantum information processing.

Until now, two main kinds of experiments have been performed to obtain information about g-factors in hybrid perovskite materials. The first includes magneto-luminescence and magneto-absorption experiments, which give access to an effective exciton g-factor that contains electron and hole contributions according to the relation g_x_ = g_e_ + g_h_ [23,24,25,26,27,28]. The second kind of experiment providing information on Landé factors includes photo-induced Kerr and Faraday rotations [29], both using a pump-probe configuration in reflectivity or transmission mode, respectively. Recently, by using these experimental techniques with a magnetic field applied in Voigt configuration (the magnetic field direction is perpendicular to the pump beam propagation direction), the $g_{⊥}$ for electrons and holes have been determined in bulk CsPbBr_3_ [14], FA_0.9_CsPbI_2.8_Br_0.2_ [30], and in CH_3_NH_3_PbI_3_ polycrystalline films [13,15].

In this article, we study the coherent evolution of the electronic spin in an oblique magnetic field by using a picosecond-photo-induced Faraday rotation (PFR) technique. We measure an unexpected anisotropy of the electron and hole Landé factors in a polycrystalline film of CH_3_NH_3_PbI_3_ (MAPI) and we discuss the possible origins of the observed anisotropy.

## 2. Materials and Methods

The samples studied here are polycrystalline films of CH_3_NH_3_PbI_3_ prepared from a solution of methylammonium iodide (1.5 mmol, 238.5 mg) and lead iodide (1.5 mmol, 691.5 mg) in γ-butyrolactone (2 mL). The new quartz substrate was cleaned in a solution of ethanol in an ultrasonic bath for 10 min and then treated with O_2_ plasma for 20 min. In a glove box, 50 μL of MAPI solution was deposited on the substrate by spin coating at 2000 rpm for 30 s. Then, the sample was annealed at 120 °C for 1 min [31]. The thickness of the resulting film was, on average, equal to 170 nm and exhibited a rough surface.

**Figure 1:**
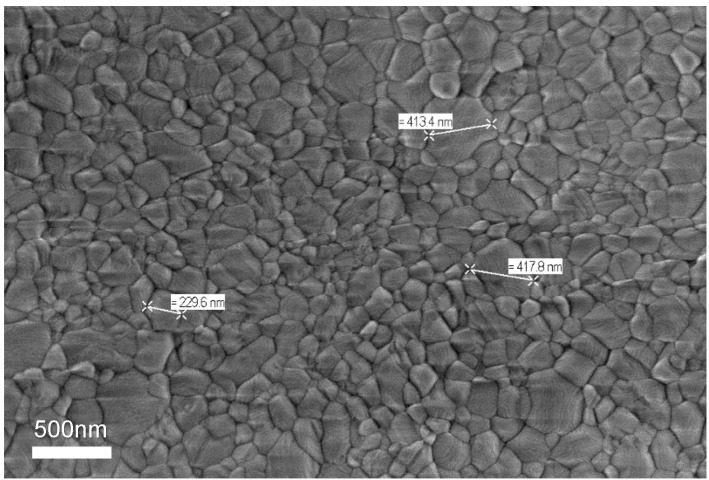
SEM image of MAPI polycrystalline films used in this study.

Film morphology was studied with a Zeiss Merlin VP Compact field emission scanning electron microscope (SEM, Zeiss Jena, Germany). The polycrystalline film constituted of grains presenting polydispersity in their sizes, from several tens of nanometers up to 400 nm, as shown in Figure 1.

**Figure 2:**
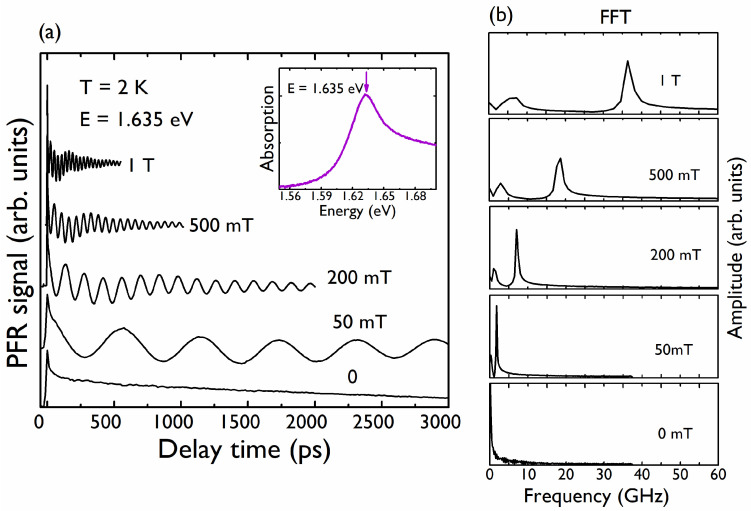
(**a**) PFR signals at 2 K for different magnetic fields. The curves have been shifted for clarity. The magnetic field is perpendicular to the propagation direction of the pump and probe beams (α = 0°). Inset: Absorption spectrum on MAPI sample obtained at 11 K. The arrow denotes the energy at which the PFR measurements were performed; (**b**) FFT of the PFR signals appearing in (**a**).

The absorption spectrum of the MAPI sample at 11 K is shown in the inset of Figure 2a. The evolution in temperature of the lower absorption peak is consistent with the orthorhombic-tetragonal transition shown by other authors [15,31].

To perform photo-induced Faraday rotation (PFR) experiments, a picosecond Ti: Sapphire laser (76 MHz repetition rate) was split into pump and probe beams. The mean optical powers were 500 μW for the pump beam and 50 μW for the probe beam; the 3-ps duration of the laser pulses allowed a spectral resolution of 1 meV. The pump beam polarization was σ+/σ− modulated at 500 kHz with an electro-optic modulator in order to avoid nuclear spin polarization. The probe beam was linearly polarized, and its intensity was modulated with an acousto-optic modulator at 3 kHz. After transmission through the sample, the probe beam was spatially resolved into its vertical and horizontal components, and the difference in intensity was measured in a balanced optical bridge. To improve the signal-to-noise ratio, a double lock-in amplifier was used for the measurements; the data presented in this work correspond to one single scan of the pump–probe delay, and thanks to the high signal-to-noise ratio (~100), no averaging of several scans is needed. The sample was placed in a cryostat containing superconducting coils. The spot size of the pump (probe) beam on the sample had a diameter of about 30 μm (15 μm), and several crystalline grains (sizes evidenced in Figure 1) on the films were thereby addressed.

## 3. Results

Figure 2a shows the PFR signal obtained at 2 K; the pump–probe energy is tuned to the maximum of the lowest absorption band of MAPI, centered at 1.635 eV. A magnetic field *B*, was applied in the Voigt configuration, i.e., perpendicular to the pump and probe propagation directions. The PFR signal contained two oscillatory contributions, which were revealed by the Fast Fourier Transform (FFT), as shown in Figure 2b. Recently, the controversy regarding the identification of these two signals has been resolved in favor of the localization of electrons and holes in CH_3_NH_3_PbI_3_ at spatially separated locations [15]. This interpretation has also been adopted and demonstrated in other halide perovskite bulk materials [14,15,30]. At zero magnetic field, the PFR curves can be described by the sum of two exponential contributions. The initial fast decay (≈ tens of ps) is likely related to the spin-polarized population of excitons bound to donors and/or acceptors and neglected in the fitting procedure (Figure 2a). The long decay of the PFR signal is related to electrons and/or holes bound to donors and acceptors. In the presence of an applied magnetic field and with a weak excitation density, the PFR signal can be well described by the sum of two damped oscillations associated with localized electrons and holes, respectively:

(1) $$\theta_{F}=A_{e}e^{-t/T_{2,e}^{*}}\cos\left(2\pi tf_{e}\right)+A_{h}e^{-t/T_{2,h}^{*}}\cos\left(2\pi tf_{h}\right),$$

where $f_{e}$, $f_{h}$ are the electron and hole frequencies, respectively, identified in the FFT, and $T_{2,e}^{*}$, $T_{2,h}^{*}$ are the corresponding dephasing times at the given magnetic field. $T_{2,e}^{*}$, $T_{2,h}^{*}$ shorten with an increasing magnetic field due to the inhomogeneities of g values [29].

**Figure 3:**
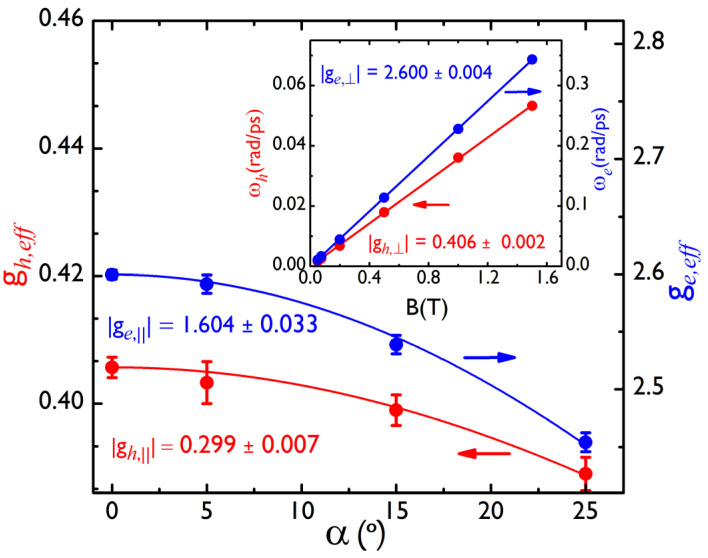
Effective Landé factors for the hole (left, red color) and the electron (right, blue color) versus the angle α. The points represent the experimental data, and the solid lines are fits to Equation (3). Inset: Larmor precession frequencies of the hole (left, red line) and the electron (right, blue line) versus the magnetic field, when the sample is perpendicular to pump and probe beams (α=0°) with an in-plane magnetic field.

We fit Equation (1) to all the PFR curves. The inset in Figure 3 shows the dependence of the two extracted frequencies $\omega_{i}=2\pi f_{i}$ (*i = e, h*) on the applied magnetic field B. The two frequencies increase linearly with the magnetic field, following the expression:

(2) $$\omega_{i}=\frac{\left|g_{i,⊥}\right|\;\mu_{B}}{\hbar }B,$$

where $\mu_{B}$ is the Bohr magneton, ℏ  the reduced Planck constant, $g_{i,⊥}$ is the Landé factor of the corresponding species, and ⊥  means that the magnetic field is applied in the plane of the film and perpendicular to the exciting light. We underline that in the full magnetic range, even for the smallest applied magnetic fields, the electron and hole Zeeman splittings show a linear behavior with the field. We conclude then that the Rashba effect is negligible in our case. From the slopes of the two linear fits, we obtained two Landé factors $\left|g_{e,⊥}\right|=2.600\;\pm \;0.004\;$ and $\left|g_{h,⊥}\right|=0.406\pm \;0.002$. We did not detect any difference in the measured Landé factors when the magnetic field was applied along two perpendicular directions in the plane of the film. These values are very close to the values $\left|g_{e,⊥}\right|=2.63$ and $\left|g_{h,⊥}\right|=0.33$ obtained in MAPI films in a recent work [13].

**Figure 4:**
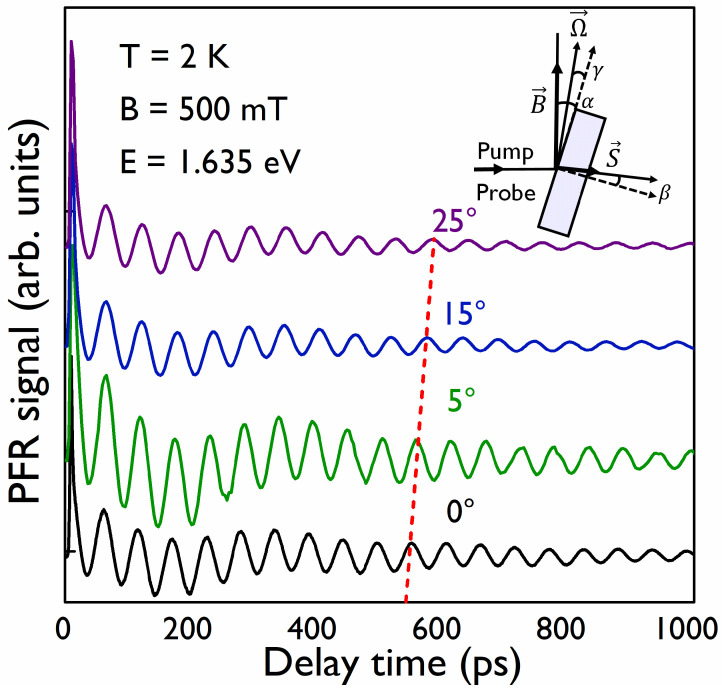
PFR signals obtained at 2 K and at *B* = 500 mT for different values of α angle. The red dashed line follows the 10-th maximum peak of the high-frequency component, showing the increase of the period as the angle α increases.

**Figure 5:**
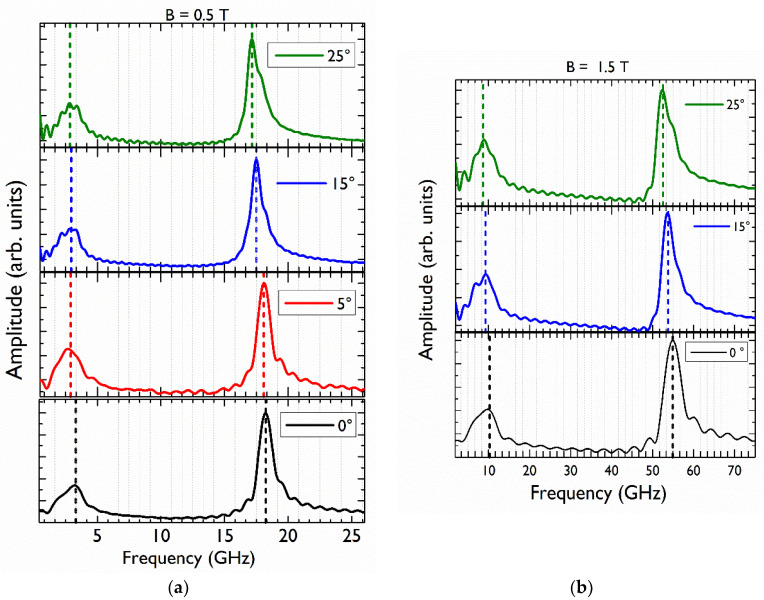
(**a**) FFT of the PFR signals appearing in Figure 4 obtained at a fixed magnetic field *B* = 0.5 T and different angles 0° (black), 5° (red), 15° (blue), 25° (green). The curves have been vertically shifted for clarity. The vertical dashed lines indicate the two identified frequencies corresponding to the hole and electron in each case. (**b**) FFT of the PFR signals obtained at *B* = 1.5 T. The decrease in the frequencies as the angle increases is indicated with the dashed lines.

To obtain a configuration for which an oblique magnetic field was applied to the sample, the sample was rotated around the axis perpendicular to the plane defined by the applied magnetic field and the pump–probe propagation direction. The rotation angle α was measured between $\vec{B}$ and the MAPI film plane. β is the angle (when α≠0) between the normal direction of the MAPI film and the direction of the oriented electronic spin, given by $\vec{S}$ as seen in the inset of Figure 4. For an anisotropic g tensor, the spin precession vector $\vec{\Omega }$ is generally non-collinear with $\vec{B}$. The vector $\vec{\Omega }$ is tilted away from the direction of $\vec{B}$ by the angle γ given by $g_{⊥}\tan\gamma =g_{∥}\tan\alpha$ with $g_{∥}$ the Landé factor for a magnetic field applied parallel to the exciting light and perpendicular to the film. Figure 4 shows PFR measurements at *B* = 500 mT for α = 0°, 5°, 15°, 25°. The period of the two contributions to each PFR signal increases as α increases. In Figure 4, this is especially clear for the high-frequency contribution (electron contribution), as shown by the red dashed line. Figure 5a shows the FFT of PFR signals shown in Figure 4. Due to the smaller anisotropy of the hole Landé factor, in comparison with the one found for the electron, it is more difficult to appreciate the decrease in the frequency (increase of the period) for the hole component in the PFR signal at only one value of the magnetic field; that is why we show, in Figure 5b, the FFT of PFR signals for different values of α at 1.5 T.

The effective $g_{i,eff}$ (*i* = electron or hole) factors for a tilted magnetic field, when α≠0, are given by:

(3) $$\left|g_{i,\;eff}\right|=\sqrt{g_{i,⊥}^{2}\cos^{2}\alpha +g_{i\;∥}^{2}\sin^{2}\alpha }\;$$

We determined $\left|g_{i,\;eff}\right|$ at each explored value of α, thanks to a linear fit of the oscillation frequency versus the magnetic field, as we have done for $\left|g_{i,⊥}\right|$. The values found for the effective Landé factor for the electron and hole versus the tilted angle, α, are plotted in Figure 3. We finally obtain, through a fit to Equation (3), $\left|g_{h,∥}\right|=0.299\pm \;0.007$ for the hole parallel g-factor and $\left|g_{e,∥}\right|=1.604\pm \;0.033$ for the parallel electron Landé factor. Very comparable results were observed on two different samples and on different points of each sample, making the results very reproducible.

## 4. Discussion

The method used to obtain the experimental values for the Landé factors does not allow us to give the sign of the g-factors, but **k.p** calculations [32] and recent experimental results [33] allow us to attribute a negative sign to the hole Landé factor and a positive sign to the electron factor.

The anisotropy ratio, defined as |gi,⊥−gi∥||gi,⊥+gi∥| and measured here, is equal to 24% for the electron and 15% for the hole. This anisotropy was fully unexpected for a polycrystalline film if all the **c** axis of the crystals were randomly oriented.

There are very few studies reporting experimental g-factor values for electrons and holes in halide perovskite materials [13,14,15,30,33]. Very recently [33], experimental results on MAPI single crystals were obtained showing a strong 43% anisotropy for the hole g-factor $\left(–0.71\leq g_{h}\leq –0.28\right)$ and a smaller 10% anisotropy for the electron g-factor $2.46\leq g_{e}\leq 2.98$. However, using these results as an experimental reference for MAPI single crystals is not straightforward, as the authors underlined that the main axes of the g-factor tensor are titled with respect to cubic axes; they gave different explanations for this experimental evidence, including the possibility of a transition to a monoclinic phase.

The already cited **k.p** calculations [32] determined anisotropic hole and electron Landé factors for the MAPI tetragonal crystal phase as follows: $g_{h,∥}=-0.472$, $g_{h,⊥}=-0.354$, and $g_{e,∥}=1.67,\;g_{e,⊥}=2.281$. In this case, the anisotropy is a consequence of the anisotropic crystal phase, and ∥ (⊥) means that the magnetic field is parallel (perpendicular) to the crystal c-axis of the tetragonal phase. This calculated anisotropy is very close to (smaller than) the experimentally observed hole (electron) g-factor anisotropy in the MAPI polycrystalline sample studied here, but the origin of the latter anisotropy in our samples has not yet been found. Among the possible causes of this anisotropy, we cite the non-random distribution of the crystallographic axes of MAPI crystals in the film or the residual strains.

A first explanation may be found in a preferential orientation of the polycrystals. Indeed, T. M. Brenner et al. [34] evidenced the preferential growth and crystallographic orientation of MAPI grains obtained from PbI_2_ crystal-precursor by using a ”two-step” synthesis method. L. Oesinghaus et al. [35] also demonstrated a preferential orientation of MAPI crystals in a polycrystalline film by using wide-angle X-ray scattering. They found a weaker preferential orientation in films obtained by ”one-step” methods than in films resulting from the use of a ”two-step” synthesis process.

On the other hand, recent studies of polycrystalline films of halide perovskites using X-ray diffraction techniques [36] evidenced the presence of lattice strains in these films due to the thermal expansion mismatch between the perovskite material and the substrate for a large variety of substrates. Cheng Zhu et al. [37], by using depth-dependent grazing incident X-ray diffraction measurements, also showed the existence of a gradient distribution of the in-plane strain component perpendicular to the substrate of halide perovskite films. Beyond the effect of these residual strains on the stability of perovskite films under illumination or on the carrier dynamics, they may also be at the origin of an anisotropy of the magnetic properties via the anisotropy of g-factors.

In conclusion, our PFR experimental study of the electron and hole spin dynamics in polycrystalline MAPI films, performed at 2 K, made it possible to measure the absolute values of the corresponding Landé factors in the orthorhombic phase. By controlling the tilt angle of the sample in the direction of the magnetic field, we observed a sharp and intriguing anisotropy of these Landé factors: 24% and 15% for the electron and the hole, respectively. At present, the origin of this anisotropy has not been identified. Further and methodical studies on X-ray diffraction must be done to clearly establish a correlation between the morphology of the polycrystalline films and the anisotropy of g-factors.

## Data Availability

The data presented in this study are available on request from the corresponding author.
